# Association between axial length and level of education in elderly patients with cataracts unexposed to electronic devices in the first two decades of life

**DOI:** 10.5935/0004-2749.2021-0294

**Published:** 2023

**Authors:** Roberto dos Reis, Rodrigo Pessoa Cavalcanti Lira, Mathias Violante Mélega, Gabriel Guerra Cordeiro, Mauricio Abujamra Nascimento, Mônica Alves, Carlos Eduardo Leite Arieta

**Affiliations:** 1 Ophthalmology Department, Faculdade de Ciências Médicas, Universidade Estadual de Campinas, Campinas, SP, Brazil; 2 Ophthalmology Department, Center of Medical Sciences, Universidade Federal de Pernambuco, Recife, PE, Brazil

**Keywords:** Axial length, eye, Myopia, Biometry, Cataract, Educational status, Humans, Aged, Comprimento axial do olho, Miopia, Biometria, Catarata, Nível de escolaridade, Humanos, Idoso

## Abstract

**Purpose:**

To determine whether the axial length is associated with the education level in elderly patients with cataracts who were not exposed to electronic devices in the first two decades of life.

**Methods:**

This cross-sectional study was conducted in elderly patients with cataracts in Campinas, Brazil. Patients were divided into 2 groups: Group 1 included those who completed, at most, elementary school (including the illiterate and those who partially or totally attended elementary school), which corresponded to 12 years of schooling; Group 2 included, at least, high school graduates (including those who completed high school and those who partially or fully attended university). The sample was selected randomly with stratification for sex and age. The main outcome was the axial length.

**Results:**

The sample consisted of 472 elderly patients (236 per group) who underwent cataract surgery. There were 272 (57.6%) men and 200 (42.4%) women; the distribution was symmetrical between the two groups. The median age (IQR; range) was 66 (12; 50-89) years. The median axial length (IQR; range) was 22.82 (1.51; 20.34-28.71) mm in Group 1 and 23.32 (1.45; 20.51-31.34) mm in Group 2 (p<0.001).

**Conclusion:**

A greater axial length was associated with a higher level of education in elderly patients with cataracts, suggesting that myopization is related to an increase in activities requiring near-vision even before exposure to electronic devices.

## Abstract (pt)

**Objetivo:**

Determinar se o diâmetro axial está asso­ciado ao nível educacional em pacientes idosos com catarata que não foram expostos a dispositivos eletrônicos nas duas primeiras décadas de vida.

**Métodos:**

Este estudo transversal foi conduzido em pacientes idosos com catarata na cidade de Campinas, Brasil. Os Pacientes foram divididos em 2 grupos: no Grupo 1 foram incluídos aqueles que completaram, pelo menos, o ensino fundamental (incluindo analfabetos e aqueles com ensino fundamental completo ou incompleto), o que corresponde a 12 anos de escolaridade; no Grupo 2 foram incluídos indivíduos que, pelo menos, estudaram até o ensino médio (incluindo indivíduos com ensino médio completo e superior completo ou superior incompleto). A amostra foi selecionada aleatoriamente com estratificação por sexo e idade. O desfecho principal foi a medida do diâmetro axial.

**Resultados:**

A amostra foi constituída por 472 indivíduos que foram submetidos a cirurgia de catarata. Duzentos e trinta e seis indivíduos (50%) foram alocados no Grupo 1 e duzentos e trinta e seis indivíduos (50%) no Grupo 2. A mediana da idade (IIQ; intervalo) foi 66 (12; 50-89) anos. Duzentos e setenta e dois (57,6%) eram homens e duzentos (42,4%) mulheres, com distribuição simétrica entre os dois grupos. A mediana do diâmetro axial (IIQ; intervalo) foi 22,82 (1,51; 20,34-28,71) mm no Grupo 1 e 23,32 (1,45; 20,51-31,34) mm no Grupo 2 (p<0,001).

**Conclusão:**

Maiores medidas de diâmetro axial foram asso­ciadas a níveis educacionais mais elevados em pacientes idosos submetidos a cirurgia de catarata. Tal achado sugere que a miopização relacionada ao aumento de atividades que utilizam a visão de perto é fenômeno que ocorre antes mesmo da exposição a dispositivos eletrônicos.

## INTRODUCTION

The axial length (AL) is an important anatomical parameter in ophthalmology and a variable for refractive errors^(1)^. According to previous studies, the AL is strongly correlated with the state of refraction^(2,3)^; for each additional 0.1 mm in AL, the eye becomes approximately 0.3 diopters more myopic.

Both the AL and incidence of myopia have been gradually increasing in recent decades^(4)^. Subjects with a higher level of education, those with occupations requiring near-vision (such as reading, writing, computer using, and video games), and those with a higher income are more likely to have a longer AL and more myopic refractions^(2,5)^. The AL drastically changes during the first 20 years of life; the eye grows from 16.5 mm at birth to about 23.75 mm in young adults. As the AL remains constant in adults^(6-8)^, the educational level in the first two decades of life may influence the development of myopia in adulthood.

Currently, there are no available data regarding the relationship between AL and education level in Brazil that utilized optical biometry. This study evaluated the influence of educational level on the AL of elderly patients with cataracts who were not exposed to electronic devices in the first two decades of life.

## METHODS

### Patients

This cross-sectional study was conducted on elderly patients with cataracts between 2016 and 2019 at the State University of Campinas (UNICAMP) in Campinas, Brazil.

The inclusion criteria were as follows: individuals over 49 years old with cataracts who were not exposed to electronic devices (i.e., smartphones, computer screens, tablets, video games) during the first two decades of life. The exclusion criteria were the following: patients who were unable to follow instructions for examinations, presence of corneal pathologies, presence of retinal pathologies that interfered with biometrics (e.g., previous retinal detachment), and patients with an abnormal eye shape (e.g., scleral staphyloma).

Patients were divided into 2 groups according to their level of education: Group 1 included those who completed, at most, elementary school (including the illiterate and those who partially or totally attended elementary school) which corresponded to 12 years of schooling; and Group 2 included, at least, high school graduates (including those who completed high school and those who partially or fully attended university). The sample was selected randomly with stratification for sex and age.

### Procedures

During the preoperative exam, the AL of the patients was measured using optical biometrics (LenStar^®^ LS900, Haag-Streit Diagnostics, Switzerland). Measurements were performed by a biometer in standard automatic mode. The subjects were then asked to blink before each scan to be acquired. If it was not possible to determine any parameter in the first capture, a second capture was performed.

No further attempts were made. Medical records and preoperative exam data were collected from each patient. Preoperative refractive error was not considered because it was not reliable due to the presence of nuclear or cortical lens changes alone or in combination with posterior subcapsular opacities.

The main outcome was to compare the AL between the two educational level groups.

### Statistical analysis

A sample size of at least 200 patients per group was determined, assuming that the study would have a power greater than 90% and a probability of type 1 error less than 0.05% (two-tailed) to detect a difference of 0.5 mm in the AL (with standard deviation of 1.5 mm and alloca­tion radius of 1) between the two groups.

The data were summarized using descriptive statistics. The Kolmogorov-Smirnov normality test was used to assess the distribution of continuous data. Medians and interquartile ranges (IQR) were used for data with non-normal distribution. The differences in continuous variables between the two groups were compared using the Mann-Whitney U test, whereas those in categorical variables were compared with the Chi-square test. Analyses were performed by SPSS version 21 (IBM Corporation, Armonk, NY, USA). The p values were two-tailed, and statistical significance was set at 0.05.

### Ethics statement

All study procedures were performed in accordance with the tenets of the Declaration of Helsinki after approval by the Ethics Research Committee of the UNICAMP (CAAE nº 45215021.7.1001.5404).

## RESULTS

The sample consisted of 472 elderly patients (236 per group) who underwent cataract surgery. There were 272 (57.6%) were men and 200 (42.4%) women; the distribution was symmetrical between the two groups. The median age (IQR; range) was 66 (13; 50-89) years in Group 1 and 66 (13; 50-88) years in Group 2 (p=0.450). The median age (IQR; range) was 67 (11; 50-89) years for men and 65.5 (16; 50-88) years for women (p=0.866).

The median AL (IQR, range) in all patients was 23.15 (1.27; 20.34-31.34) mm. The median AL (IQR; range) was 22.82 (1.51; 20.34-28.71) mm in Group 1 and 23.32 (1.45; 20.51-31.34) mm in Group 2 (p<0.001). The median AL (IQR; range) was 23.34 (1.52; 20.64-31.34) mm in men and 22.76 (1.45; 20.34-28.71) mm in women (p<0.001). Table 1 displays the AL data according to the educational level (Group 1 vs. Group 2) stratified by sex.

**Table 1:** Axial length according to educational level (Group 1 vs. Group 2) stratified by sex

Sex	Group 1	Group 2	p
**Male** ^a^	23.13 (1.53; 20.64-26.60)	23.50 (1.30; 21.17-31.34)	0.002^b^
**Female** ^a^	22.33 (1.23; 20.34-28.71)	23.15 (1.37; 20.51-26.96)	<0.001^b^

a = median (IQR; range) mm;

b = Mann-Whitney U test comparing patients in Group 1 vs. Group 2; Group 1 included those who completed, at most, elementary school (including the illiterate and those who partially or totally attended elementary school), corresponding to 12 years of schooling; Group 2 included, at least, high school graduates (including those who completed high school and those who partially or fully attended university).

## DISCUSSION

This is the first Brazilian study that utilized optical biometry to provide evidence of a positive association between the AL and educational levels. A higher AL was associated with a higher level of education in elderly patients with cataracts who were not exposed to electronic devices during the first two decades of life. These findings were observed in both sexes.

The association of a longer AL with a higher education level in our study is in agreement with the results of previous population-based studies^(1,2,4,6,9-11)^. Regarding sex variations, we observed that women had shorter eyes than men, which was already reported by other authors^(1-3,7,8,10,12)^.

Exposure to lifestyle changes resulting from a combin­a­tion of reduced outdoor time and increased near-vision work activities in the early years lead to refractive error and myopia^(2,4,5,10,13)^. The mechanism for myopic refractive error appears to be axial elongation. Among environmental factors, the so-called high-pressure education system, especially at young ages, can be a causative lifestyle change as well as the excessive use of electronic devices such as smartphones, tablets, and video games^(2,13)^. Palliative measures in the ocular growth phase (childhood and adolescence), such as regular breaks during activities requiring near-vision (e.g., reading), increase in outdoor activities, and reduction of children’s screen time, and pharmacological measures (atropine eye drops in low concentrations) in selected cases seem capable of counteracting the increased risk of myopia^(14-16)^. However, the incidence of axial myopia has increased over the last few decades^(17)^. Therefore, strategies aimed at reducing the prevalence of high myopia should be considered, since complications associated with this ocular pathology have already been reported^(18)^.

In our study, having a higher level of education was a strong determinant for a longer AL; as the patients were born between 1930 and 1970, they were not exposed to electronic devices during childhood and adolescence.

The following limitations must be considered. First, the relationship between biometric characteristics and refraction has not been evaluated due to lens opacification in these patients. Second, there were no data available on outdoor activities. Finally, we cannot exclude selection bias because the study was clinic-based and may not be representative of the entire population.

In conclusion, a longer AL was associated with a higher level of education in elderly patients with cataracts, suggesting that myopization was related to an increase in activities requiring near-vision, which existed even before exposure to electronic devices.
